# Comprehensive Assessment from Optimum Biodiesel Yield to Combustion Characteristics of Light Duty Diesel Engine Fueled with Palm Kernel Oil Biodiesel and Fuel Additives

**DOI:** 10.3390/ma14154274

**Published:** 2021-07-30

**Authors:** Senthur Prabu Sabapathy, Asokan Morappur Ammasi, Esmail Khalife, Mohammad Kaveh, Mariusz Szymanek, Gokul Kuruvakkattu Reghu, Prathiba Sabapathy

**Affiliations:** 1 School of Mechanical Engineering, Vellore Institute of Technology (VIT), Vellore 632014, India; senthurprabu.s@vit.ac.in (S.P.S.); gokul20000@gmail.com (G.K.R.); 2 Department of Civil Engineering, Cihan University-Erbil, Kurdistan Region, Iraq; esmail.khalife@su.edu.krd; 3 Biofuel Research Team (BRTeam), Terengganu, Malaysia; 4 Department of Biosystem Engineering, Faculty of Agriculture and Natural Resources, University of Mohaghegh Ardabili, Ardabil 56199-11367, Iran; sirwankaweh@gmail.com; 5 Department of Agricultural, Forest and Transport Machinery, University of Life Sciences in Lublin, Głęboka 28, 20-612 Lublin, Poland; 6 Department of Chemical Engineering, SSN College of Engineering, Chennai 603110, India; prathibabiochm@gmail.com

**Keywords:** biodiesel, diesel engine, combustion, transesterification, BHT, DEE

## Abstract

Biodiesel is considered as a key prospective renewable energy source in India. Hence, a study was carried out for the improvement of palm kernel oil biodiesel production using a transesterification process at different molar ratios. This study comprehensively examined all aspects of biodiesel from optimum production to the effect of additives on its combustion behavior. The optimum yield condition was validated with the MINITAB-17 software and analyzed using the Taguchi method. Two different additives, 5% diethyl ether (DEE) and 2000 ppm Butylated hydroxyltoluene (BHT), were also experimented. Engine experiments were conducted at constant speed (1500 rpm) and five different engine loads (0, 25, 50, 75 and 100%) on a single-cylinder direct injection diesel engine. Heat release rate, brake specific fuel consumption, brake thermal efficiency, engine emissions, such as CO, HC, NOx, and smoke opacity were analyzed. The maximum palm kernel oil (PKO) biodiesel yields, obtained at 55 °C, for the KOH and NaOH catalysts were 86.69% and 75.21% at the molar ratio of 6:1. B20BHT combustion showed 4.6% higher brake thermal efficiency (BTE). NOx emission was reduced by 19.4%, compared to the diesel fuel values. DEE resulted in higher CO and HC emissions compared to diesel fuel values by 39.2% and 7.6%, respectively, whereas smoke emission was improved by 11.5%.

## 1. Introduction

In the current scenario, the world energy resources are quickly depleting every year due to the rising trend of industrialization, as well as modernization. Crude oil is the main source to fulfill the demand of energy, but it leads to environmental degradation. Moreover, the products of combustion, such as NOx, SO_2_ and CO_2_, result in global warming. The atmosphere problems caused by the consumption of fossil fuels alongside petroleum scarcity have led researchers to explore renewable energy resources, i.e., derivatives of animal fats, vegetable oils and waste oils. Since the previous century, the thought of vegetable-oil-based fuel (biodiesel) to operate diesel engines has been on the world stage. Biodiesel as a substitute fuel has been getting great attention among the world policy makers due to its positive properties like low exhaust gases emissions, non-toxic characteristics and biodegradability, as well as renewability [1,2]. This fuel is used in compression ignition (CI) engines with no or slight modification. However, due to the greater viscosity, flash point, pour point, etc., vegetable oils must be transesterified [3], that is, the triglyceride molecules are cracked into methyl ester (biodiesel). Lipids react with alcohol to form esters and a by-product, glycerol. For this aim, methanol and ethanol are widely used alcohols. Biodiesel blended with diesel has become very popular, nowadays, because of its increased lubricity, which reduces wear and tear of engine components and increases safety. while storing and transporting of biodiesel are effortless due to its high flash point and its compatibility in blending with diesel fuel. However, biodiesel has some limitations, such as clogging, due to its greater viscosity, high flash point and higher NOx emissions, compared to diesel fuel [4,5,6]. Therefore, it is essential to refine biodiesel to obtain better quality and ensuring about its performance and emissions. Low acid value, higher cetane number, low viscosity, low specific gravity and high flash point are some of the major factors for the quality of biodiesel [7,8]. Among all biodiesel resources, non-edible oils are more interesting than edible oils because of competition between food and energy. In that aspect, palm kernel oil (PKO), as non-edible oil, can be conceived as potential inexpensive biodiesel feedstock and as an alternative compared with the new non-edible/edible oils. The main advantage of PKO is its ready availability in India and it is being reused for the production of biodiesel [9,10]. There are several papers on converting PKO into biodiesel. For example, Alamu et al. [11] investigated biodiesel production through transesterification of PKO with ethanol and in the presence of NaOH as catalyst and glycerol as a byproduct. They reported a high yield biodiesel production of up to 96%. Beside the method of biodiesel production, there are several parameters which impact biodiesel production yield. For instance, Aboelazayem et al. [12] studied very important variables which influence biodiesel yield, such as molar ratio, catalyst concentration, temperature, stirring rate and time, to maximize biodiesel production from castor oil. They found that molar ratio has a directly proportional relationship with biodiesel yield in a range of 3:1–7:1. However, further increasing molar ratio has no valuable effect on biodiesel yield. A similar observation was reported by Silitonga et al. [13] in their study, where they concluded that the effect of molar ratio has a highly statistical significant effect on biodiesel yield in a molar ratio range of 3:1–6:1. They reported that, at an M:O molar ratio higher than 9:1, the increase of M:O molar ratio has a negative effect on biodiesel yield, resulting in decreasing it. PKO biodiesel combustion was investigated from many aspects. Ayetor et al. [14] studied the performance and emission characteristics of PKO biodiesel–diesel blends in a direct injection (DI) diesel engine and reported that the emission characteristics of HC and CO of B100 were reduced by 55% and 41%, whereas NOx increased by 10%, in comparison with diesel fuel values. They also reported 6% increase for BSFC. Nwakaire et al. [15] studied engine performance with different ratios of biodiesel blended in diesel fuel and claimed an improvement, compared to diesel fuel, of up to 26%; in addition, they reported low BSFC for biodiesel combustion. However, one of the major problems with biodiesel application as fuel is its oxidation stability. For this aim, many studies have focused on the effect of antioxidants on biodiesel properties and combustion. For instance, Prabu et al. [16] studied the effect of Butylated hydroxyltoluene (BHT) and n-butanol on diesel engine emission parameters fueled with diesel–waste cooking oil (WCO) biodiesel blends. They investigated that the inclusion of BHT into B30 resulted in higher brake specific fuel consumption (BSFC), by 7.3%, and lower BTE, by 4.6%, compared with diesel, whereas the heat release rate (HRR) was almost comparable to diesel fuel. In addition, the oxygenated content of n-butanol added to the B30 blend showed a CO emission reduction of 37.5% and a NOx emission increment of 9%, compared to diesel. In an interesting investigation, Ryu [17] considered the effect of different antioxidants on biodiesel oxidation stability, as well as diesel engine combustion parameters, and reported that the antioxidants efficiency is in the order of α-tocopherol < BHT < BHA < PrG < TBHQ. They pointed out that all of the antioxidants had lower BSFC than neat biodiesel fuel. Ndayishimiye and Tazerout [18] carried out a diesel engine test using palm oil (PO) biodiesel blended with diesel fuel to evaluate exhaust gas emission and engine performance characteristics. They reported high BSFC, increasing (2–25%) for different preheated biodiesel ratios, while BTE showed a similar trend as diesel combustion. HC emission was drastically reduced by 30–65% with preheated oil and WCO + PO biodiesel, compared to diesel. They found NOx emission was reduced with PO biodiesel, compared to diesel fuel. 

To improve some biodiesel drawbacks, such as viscosity, researchers include additives, such as diethyl ether (DEE), alcohol, etc. For example, Imdadul et al. [19] investigated the combustion of Calophyllum inophyllum biodiesel containing pentanol in a DI diesel engine and stated that the added alcohol improved the burning premixed and diffusion stages of combustion. In another study, Qi et al. [20] investigated the effect of a biodiesel–diesel (B30) blend containing ethanol (5%) and DEE (5%) on diesel engine combustion and observed that the addition of DEE led to a reduction in BSFC. They reported that the combustion of DEE additive led to higher HRR and peak pressure of the cylinder at higher engine loads. In another study, Imtenan et al. [21] tried to find the effect of n-butanol and diethyl included in diesel–jatropha biodiesel blends and stated that DEE showed lower BSFC than neat diesel at low engine speeds, which actually points out better combustion quality due to their high oxygen content and low viscosity, compared to n-butanol. Apart from viscosity, biodiesel degradation is also another important problem which influences its combustion quality. The addition of antioxidant additives into the biodiesel fuel can properly prevent from its oxidation to a reasonable level. The examination of various studies showed that almost studies have just focused on the effect of the molar ratio of the biodiesel production or have considered the biodiesel combustion behavior. To the best of the authors’ knowledge, there is no research which has targeted all aspects of biodiesel fuel from production condition to combustion, as well as the effect of additives. Therefore, the purpose of the present study was to comprehensively scrutinize the PKO biodiesel optimum production and make a comparison on combustion characteristics and exhaust gases of DEE (10 vol%) and BHT (2000 ppm) additives. Seven different biodiesel–diesel blends (pure diesel, B20, B30, B40, B100, B20DEE and B20BHT) were examined under five engine loads (no load, 20, 40, 60, 80 and 100%) and constant speed (1500 rpm) of a 4-stroke, water cooled, one cylinder DI diesel engine. 

## 2. Materials and Methods

**Figure 1:**
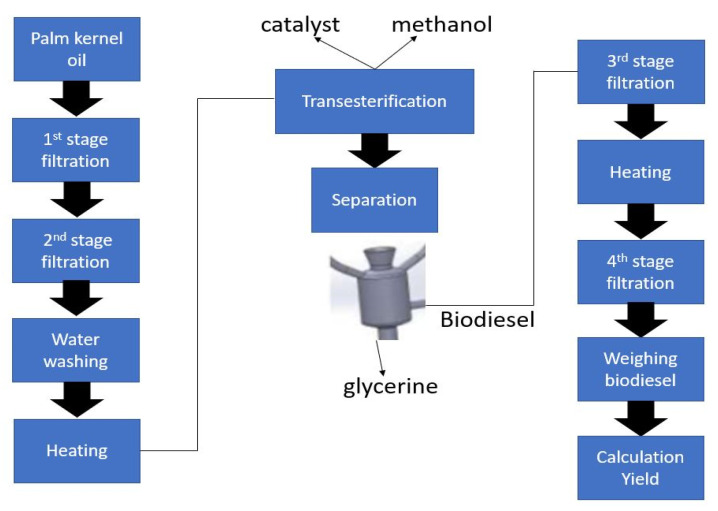
Flow chart of the transesterification process.

PKO is an available, abundant and low cost source for biodiesel production. The schematic steps of the procedure adopted for biodiesel production from PKO is illustrated in Figure 1.

### 2.1. Transesterification Process

The transesterification process is affected by the type and quantity of alcohol, time and temperature of the reaction, type and quantity of the catalyst and amount of free fatty acid (FFA). As above mentioned, ethanol and methanol are commonly and widely used alcohols. However, due to the low cost and reactivity, methanol was used for biodiesel production in the present work. The transesterification reaction was performed in two steps. First, an acid catalyzed reaction was performed using H_2_SO_4_ (sulphuric acid) in the presence of methanol; the second step was carried out using a base catalyzed reaction with potassium hydroxide (KOH) and sodium hydroxide (NaOH), separately. In the acid catalyzed reaction, triglycerides were converted into diglycerides; then, during the base catalyzed reaction, diglycerides were converted into mono glycerides and, at the end, monoglycerides into glycerol. 

The production of PKO biodiesel samples was performed through methanol at 3 different ratios, 3:1, 6:1 and 9:1 (i.e., methanol to oil ratio or molar ratio), 3 different reaction temperatures (50, 55 and 60 °C) and a reaction time of 45 min, with the H_2_SO_4_ acid catalyst in the 1st step and KOH and NaOH as base catalysts in the 2nd step. 

The PKO was filtered through a strainer, initially, to remove the large, suspended particles; 3% of orthophosphoric acid was added to the oil and left for 3 h, for settling heavy particles; then, the oil was separated using a filter paper (Watman 6: 25 µm); further, the oil was washed with distilled water. Water was then removed automatically from the bottom of the flask, because of the density difference between water and oil; however, the remaining water was removed by heating the oil to 100 °C and maintaining it for 3–4 h, otherwise, the moisture in the oil would have caused negative effects on the biodiesel yield. Thereafter, the FFA value of PKO was found using the titration method, in which the solution of oil (1ml), isopropyl alcohol (10 mL) and 2–3 drops of phenolphthalein was titrated against a KOH solution (1 kg of KOH + 1 L of water); the color change to pink was considered as the titration values. The acidic value was estimated using Equation (1) [22]:

(1) $$Acidic\;value\;\left(AV\right)=\left(v_{eq}-b_{eq}\right)N\frac{56.1\;{\mathrm{gmol}}^{-1}}{W_{oil}}$$

where, *v_eq_* is the final reading (mL), *b_eq_* the initial reading (mL), *N* the normality of KOH and *W_oil_* the oil weight (g).

FFA Value = AV × 0.52(2)

The FFA value was calculated from Equation (2). Accordingly, the amount of catalyst required for transesterification reaction was obtained through the calculating FFA value in volume basis (*v*/*v*), i.e., 1.067, 1.152 and 1.408 g for the molar ratios of 3:1, 6:1 and 9:1, for the KOH catalyst, whereas, for the NaOH catalyst, 1.152, 1.195 and 1.707 g for the molar ratios of 3:1, 6:1 and 9:1. 

A volume of 100 mL of PKO was taken in a beaker and then added into the KOH + methanol solution, which was prepared separately. Thereafter, the solution with the reaction time of 45 min was stirred using the orbital shaker at a speed of 200 rpm. Afterward, the mixture was kept for 12 h to let the glycerine settle down at the bottom of the beaker. After that, the biodiesel was washed and heated to 65 °C, to remove the traces of KOH or methanol. Finally, the biodiesel collected was filtered and weighed to find the yield, which was calculated using Equation (3) [23]:

(3) $$Yeild\;\%=\frac{Weight\;of\;biodiesel}{weight\;of\;oil}$$

The same process was repeated for each sample (100 mL) of PKO with various different alcohol ratios (3:1, 6:1, 9:1) and three different temperatures (50, 55 and 60 °C) for a reaction time of 45 min, employing KOH and NaOH, separately, as catalysts. The range and levels for different parameters are given in Table 1.

### 2.2. Biodiesel Production Model

The MINITAB-17 software was used for the statistical analysis of the experimental data and the correlation of the parameters to the biodiesel yield (%) was given by a fully quadratic model in Equation (4):

(4) $$Yield\;\left(\%\right)=a_{0}+\sum_{n=1}^{4}a_{n}x_{n}+\sum_{n=1}^{4}a_{nn}x_{n}{}^{2}+\sum_{n=1}^{4}\sum_{m=n+1}^{4}a_{nm}x_{nm}$$

where, $x_{n}$,$\;x_{nm}$ are uncoded independent variables, $a_{0}$ is a constant and $a_{n}$,$\;a_{nn}$,$\;a_{nm}$ are the regression coefficients.

### 2.3. Properties of PKO

#### 2.3.1. Flash Point and Fire Point

The flash point and fire point are important thermal properties of a fuel which give an idea of the lowest temperature where the vapors of the fuel start to ignite in the presence of a source of ignition. With the help of an apparatus called Pensky–Martens (Aimil Ltd., New Delhi, India), the flash and fire points were measured for the PKO biodiesel–diesel blends, as per the ASTM D93 standard. As can be observed from Table 2, the flash point of PKO biodiesel was higher than that of diesel fuel. As from a safety point of view, pure PKO biodiesel is a better option, compared to diesel.

#### 2.3.2. Kinematic Viscosity and Density

Kinematic viscosity is a key physical property of a fuel, which influences combustion. By using the Redwood viscometer (Aimil Ltd., New Delhi, India), the kinematic viscosity was found for the diesel–PKO biodiesel blends, as per the ASTM D 445 standard, and tabulated in Table 2. The kinematic viscosity of the diesel–PKO biodiesel blends was measured at 40 °C and it was observed that the viscosity of B100 (4.2 mm^2^/s) was higher than that of diesel fuel (2.75 mm^2^/s). However, the measured values of kinematic viscosities were inside the range of biodiesel. Further, the average density of the diesel–PKO biodiesel blends was measured at 20 °C, as per the ASTM 1298 standard, and the value of PKO was 844 kg/m^3^, whereas, for diesel, it was 830 kg/m^3^. The density of PKO biodiesel was greater, by 1.6%, than the diesel fuel one. The density of the PKO biodiesel produced was somewhat higher than that of diesel fuel; however, it was inside the range of ASTM standards.

#### 2.3.3. Calorific Value (CV)

The CV is an important thermal property of fuels which shows the amount of heat energy produced during the complete burning per unit quantity of fuel burnt. A bomb calorimeter (EIE Instruments Private Ltd., Ahmedabad, India) was used to determine the CV of diesel–biodiesel blends (ASTM D 240 standard); the results are presented in Table 2. The calculated CV of PKO was 38.2 MJ/kg, whereas, for diesel fuel, it was 43.8 MJ/kg. The CV of PKO biodiesel was approximately 12.78% lower than the diesel fuel one, which entails that the amount of biodiesel injected needs to be more to attain the same power as diesel fuel.

#### 2.3.4. Cetane Number (CN)

The cetane number (CN) is a parameter which determines the ignition delay period (the period between fuel injection and SOC). As per the ASTM D613 standard, the CN of PKO biodiesel was acquired from Bello et al. [9]; the obtained results are tabulated in Table 2. In this study, the CN of PKO biodiesel was 52, which is lower than the diesel fuel one, 53, which might slightly increase the time of ignition delay.

### 2.4. Preparing Samples

**Figure 2:**
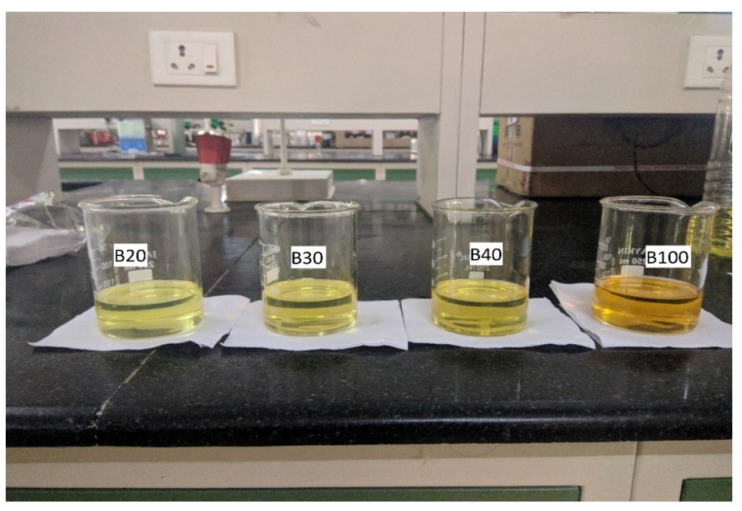
Photographical view of PKO blends.

To prepare biodiesel–diesel blend, the obtained PKO biodiesel was blended with diesel by volume, i.e., B20 (20% PKO biodiesel + 80% diesel), B30 (30% PKO biodiesel + 70% diesel), B40 (40% PKO biodiesel + 60% diesel) and B100 (100% palm kernel oil biodiesel), as shown in Figure 2. Here, pure diesel was employed as control sample. 

For preparing other blends, DEE and BHT additives were included separately with B20. DEE additive was added into B20 to prepare a B20 + DEE fuel blend by blending 100 mL of DEE in 900 mL of B20 blend. For the next samples, BHT was added into B20 to obtain a B20 + BHT fuel blend by adding 2000 ppm BHT in 1000 mL of B20.

The effects of catalyst, temperature and methanol ratio for different trials on the biodiesel yield (%) of the results obtained from the experiment and predicted by model proposed are tabulated in Table 3.

From Table 3, the maximum biodiesel yield (86.69%) using the catalyst KOH was obtained from the molar ratio of 6:1 at a reaction temperature of 55 °C, whereas the maximum yield was 88.01%, according to the proposed model. The maximum yield using the NaOH catalyst (75.21%) was obtained from the molar ratio of 6:1 at a temperature of 55 °C and, for the model generated by the software, it was 73.37%. PKO biodiesel was produced with the help of the highest yield parameters obtained (Table 3), i.e., catalyst, reaction temperature and molar ratio. The thermophysical properties of diesel–PKO biodiesel blends were obtained using ASTM D6751 standard and are represented in Table 2. The table clearly shows that the PKO biodiesel produced by transesterification process was within the range of ASTM standards.

### 2.5. Engine Combustion Setup

**Figure 3:**
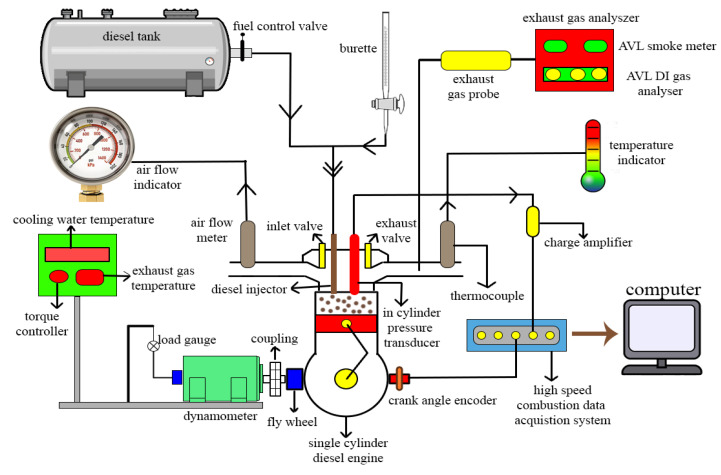
Schematic diagram of experimental engine setup.

Performance and emission characteristics of all prepared samples were carried out using a single-cylinder, 4-stroke DI diesel engine (Kirloskar, Pune, India), which was operated at a constant engine rotation of 1500 rpm. The diesel engine was paired with an eddy current dynamometer for varying loads, from 0 to 100%, in steps of 25%. A schematic of engine test setup is shown in Figure 3. In addition, the specifications of the engine are tabulated in Table 4.

An AVL gas analyzer (DIGAS 444 N model)(Indiaprivate Ltd., Hyderabad, India) was employed to measure the NOx, CO and unburned hydrocarbon (UHC) emissions of the engine. In addition, the smoke intensity was measured by an AVL 437 C smoke opacimeter. A thermocouple (K-type) was provided to evaluate the inlet air and exhaust gas temperature, cooling water and lubricating oil temperature. HRR were measured by employing an AVL 617 Indimeter V2.0 (AVL India private Ltd., Hyderabad, India). The data (position of crank angle during the start of combustion (SOC), HRR and peak pressure) were recorded and a file was generated during the test by the Indimeter software V2.0. A miniature pressure sensor AVL GH12D was employed for measuring the variation of pressure in-cylinder vs. crank angle. The encoder was used to obtain the position of the crank angle. Additionally, a piezoelectric amplifier (AVL 3066A02) was employed to intensify the output signal from the miniature sensor.

#### 2.5.1. Testing Conditions

During the initial starting of the diesel engine, the neat diesel fuel was used to operate in full throttle opening condition at a constant speed of 1500 rpm to run at least 30 min to reach a steady state condition. The lubricating oil temperature was ensured to be within the range 85–90 °C, whereas the temperature of cooling water was maintained at 60 °C. 

Before starting the engine tests of diesel–PKO biodiesel blends, the neat diesel fuel was first tested by changing loads from 0 to 100%, in steps of 25%, after it was ensured that the diesel engine reached a steady state condition. For each load, the engine was set to run at 1500 rpm. A time interval for 10 cc consumption of fuel was entered with the help of a stopwatch. The data, such as power output, in-cylinder pressure, engine speed, fuel consumption and exhaust emissions, were measured and recorded carefully. BTE and BSFC, along with the combustion and emission parameters, were computed for engine performance. Thereafter, the diesel–PKO biodiesel blends were tested and all the data were recorded for each sample simultaneously. After completing each and every test, the diesel engine was made to continue to operate till the fuel left in the fuel tank was completely drained; then, the subsequent fuel blend was poured. 

#### 2.5.2. Error Analysis and Uncertainty

Calculating the uncertainty of the equipment employed is a method to get more precise results. Because of environmental and operating conditions, calibration and selection of instrument, instrument quality and test order, etc., the results may vary and the same results could not be obtained. Experiments were iterated three times and the average values were taken for plotting the graph. Table 5 shows the uncertainties, as well as accuracies, of the equipment used in this investigation. The overall uncertainty percentage (in this study, ±2.53%) was calculated, based on the combined effect caused by the uncertainty of the various measuring instruments and propagation of errors from Holman [24], as follows. Percentage of uncertainty occurring in the experiments = root square of uncertainty of [(angle encoder)^2^ + (pressure transducer)^2^ + (NOx)^2^ + (O_2_)^2^ + (CO)^2^ + (CO_2_)^2^ + (Smoke opacity)^2^ + (HC)^2^ + (K-2 thermocouple)^2^ + (manometer)^2^ + (stop watch)^2^ + (burette)^2^] = root square of [(0.3)^2^ + (0.01)^2^ + (0.5)^2^ + (0.35)^2^ + (0.02)^2^ + (0.2)^2^ + (0.3)^2^ + (1.1)^2^ + (1.5)^2^ + (0.3)^2^ + (1.5)^2^] = root square of (6.393) = ±2.53%

## 3. Results and Discussions

### 3.1. Optimization of Biodiesel Production

**Figure 4:**
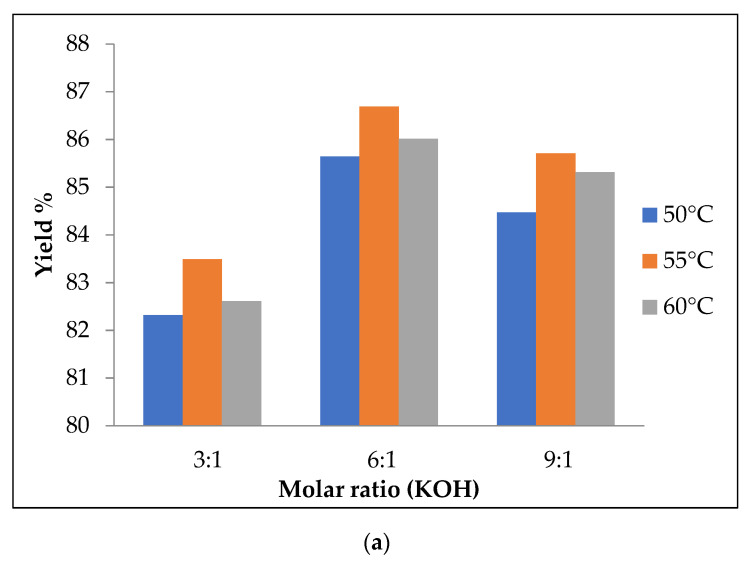

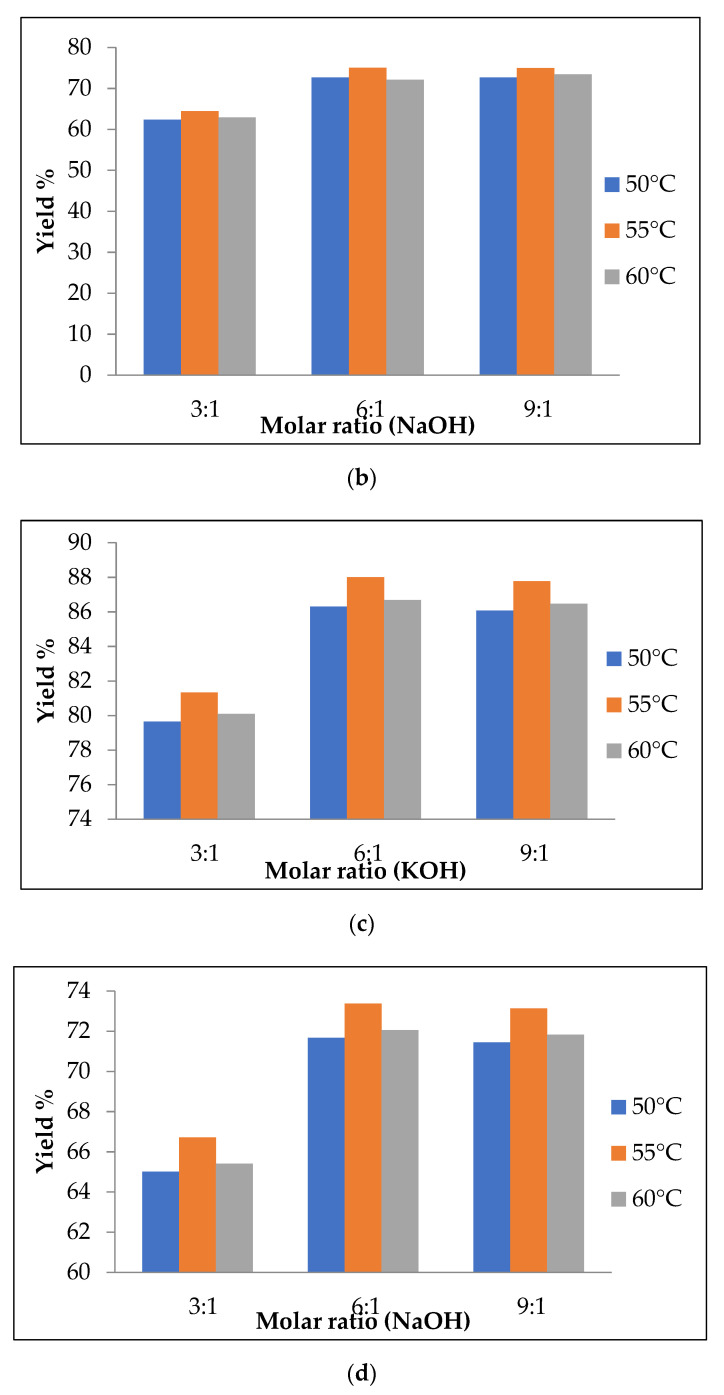
(**a**) Experimental results using the KOH catalyst. (**b**) Experimental results using the NaOH catalyst. (**c**) Modeling results using the KOH catalyst. (**d**) Modeling results using the NaOH catalyst.

**Figure 5:**
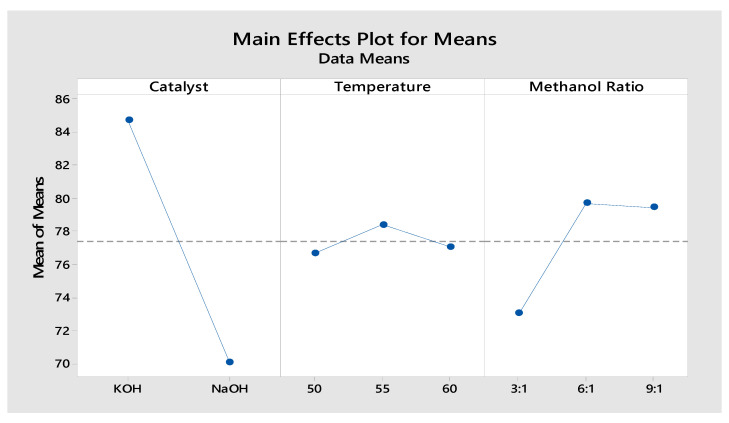
Plot of model experiment: means versus catalyst, temperature and methanol ratio.

The influence of various alcohol ratios (3:1, 6:1, 9:1), i.e., molar ratio using two different catalysts (KOH and NaOH), with 3 different reaction temperatures (50, 55, 60 °C) for a time of 45 min, on the production of PKO biodiesel yield (%) were experimentally investigated (Figure 4a,b) and validated using the MINITAB-17 software (Figure 5). The results of both experimental results and predicted values by the model proposed are tabulated in Table 3. 

#### 3.1.1. Effect of Different Ratios of Molar Ratio on PKO Biodiesel Yield

Figure 4a,b depict the obtained PKO biodiesel yield for different molar methanol-to-oil ratios for a reaction time of 45 min, using KOH and NaOH as catalyst, with 3 different temperatures (50, 55 and 60 °C). For the molar ratio of 3:1, the biodiesel yield for both KOH and NaOH catalysts was lower, due to the insufficient methanol level in the oil for the reaction. The maximum yield of PKO biodiesel was obtained at the molar ratio of 6:1 with the temperature of 55 °C (Table 3) for the KOH (86.69%) and NaOH (75.21%) catalysts. From the results, the biodiesel conversion was more effective in the methanol level of 6:1, as can be also observed from the Figure 4. The biodiesel conversion was decreased with further increase in the molar ratio (i.e., 9:1), probably because of the fact that glycerol separation from biodiesel fraction is very hard. This is due to the high glycerine solubility that affects the settling of glycerine, thereby decreasing the biodiesel yield [25]. It is observed, from Figure 4, that the KOH catalyst had a higher biodiesel yield, compared to the NaOH catalyst, due to the higher glycerol separation.

#### 3.1.2. Effect of Different Catalysts on the Biodiesel Yield

The amount of catalyst loading on the molar ratio influences the production of biodiesel by accelerating the reaction rate. The amounts of catalyst required for KOH, based on FFA values, were 1.067, 1.152 and 1.408 g at the molar ratios of 3:1, 6:1 and 9:1, respectively, whereas the amounts for the NaOH catalyst were 1.152, 1.195 and 1.707 g at the molar ratios of 3:1, 6:1 and 9:1. As observed in Table 3, KOH and NaOH influence the biodiesel production yield. The maximum PKO to biodiesel conversion (86.69%) for the catalyst KOH was obtained at the conditions of molar ratio of 6:1, temperature of 55 °C and time of 45 min. The biodiesel production was decreased by increasing catalyst concentration over the molar ratio. This might be due to the stable emulsion created because of the glycerine interaction with the excess methanol, which affects the settling of glycerine [25]. For the NaOH catalyst, the maximum biodiesel yield from PKO was obtained at the molar ratio of 6:1, temperature of 55 °C and reaction time of 45 min. At higher molar ratio, higher amount of the NaOH catalyst decreased the biodiesel yield. The reason might be because of soap formation during the reaction, which results in product losses [25]. Additionally, the biodiesel yield decreased, with the NaOH catalyst, due to greater water formation during the reaction. From Figure 4a,b, the biodiesel conversion yield was higher with KOH, compared to the NaOH catalyst. KOH could lead to 15.5% higher biodiesel yield than the NaOH catalyst at the molar ratio of 6:1, whereas, at the ratio of 3:1, the difference was 29.57%.

#### 3.1.3. Effect of Temperature on the Yield of Biodiesel Production

Various reaction temperatures, such as 50, 55 and 60 °C, were studied in the present investigation. Maximum PKO biodiesel yield was found at the temperature of 55 °C for both catalysts, due to maximum methanol diffusivity in the oil which leads to collisions among molecules, thereby allowing glycerine to settle, as observed in Figure 4. With temperature rising to 60 °C, the biodiesel yield decreased slightly, due to the evaporation of the methanol [25]. In addition, higher temperature increases saponification, compared to the transesterification reaction.

#### 3.1.4. Linear Model Analysis

The regression coefficient yield of PKO biodiesel was determined using the MINITAB-17 software, using the full quadratic the model proposed given in Equation (4). The values of the coefficient and adjusted coefficient (R^2^ and R^2^ adj.) were 95.13% and 93.10%, respectively. Since R^2^ is close to 95% the null hypotenuse is proven and the values are verified. From Table 3, the model proposed biodiesel yield variations almost matched the experimental biodiesel yield ones. The optimum condition of the model yield was 88% for biodiesel yield in the conditions of 1 wt.% KOH catalyst, 6:1 molar ratio and at 55 °C temperature, as presented Figure 4c,d whereas the optimum condition for the experimental biodiesel yield was 87%. This shows the fully quadratic the model proposed for prediction of the biodiesel yield was highly reliable.

Biodiesel yield (%) = 93.58 + 3.558 (MR) + 1.750 (C) + 2.589 (T) − 0.33 (MR^2^) − 2.800 (C^2^) − 1.280 (T^2^) + 0.238 (MR × C) + 0.008 (MR × T) + 0.66 (MR × T) + 0.192 (C × T) − 1.6(C × T) − 0.175(P × T) (5)

### 3.2. Combustion Characteristics

The performance and emissions of different blends of diesel–PKO biodiesel blends (B0, B100, B20, B30 and B40), B20 + DEE and B20 + BHT, which were tested in a single-cylinder, 4-stroke DI diesel engine at a constant speed of 1500 rpm at various load conditions, are presented in this section. The discussed combustion and performance parameters are HRR, in-cylinder pressure, BTE and BSFC and the discussed exhaust emission are CO, NOx, HC, EGT and smoke opacity.

#### 3.2.1. Heat Release Rate

**Figure 6:**
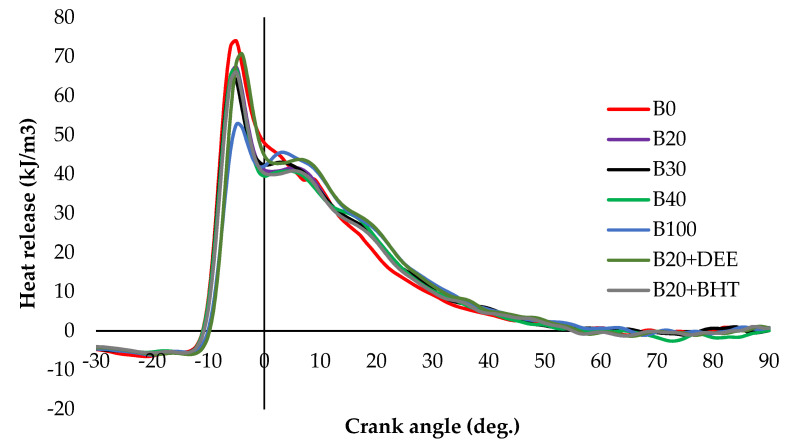
HRRs of all fuel blends at full load.

Figure 6 shows the variations in HRR of different diesel–PKO biodiesel blends. HRR is an important parameter which gives in-depth information about the burning characteristics and provides a way to calculate the combustion duration. Actually, in the DI diesel engine, during combustion, the heat release happens in two phases. The premixed combustion (PC) phase is the first phase; after the injection starts, the air/fuel-rich mixture is formed because of the delay in ignition where the pressure is raised. The HRR also gives in-depth information about the SOC, which was occurred at 12–13° before top dead center (BTDC) for all fuel tested. If fact, when the piston reaches near the TDC, the vaporization of fuel occurs, due to the high pressure and temperature. Because of the ignition delay, which causes a negative heat release, once the ignition starts, the heat release momentarily changes to positive. Once the combustion starts, the fuel-rich mixture burns rapidly and uncontrolled combustion occurs, where the heat release is at its maximum. The diffusion combustion (DC) phase is the second phase. In the diffusion phase of combustion process, improved mixing of the air and fuel takes place after the oxygen is consumed. This results in controlled combustion which happened at up to 18° after top dead center (ATDC). Then, the progressive burning happens at up to 45° ATDC and also the peak heat release occurred between −6° BTDC to 8° ATDC (as shown in Figure 5). These values, for B100, B0, B20, B30, B40, B20 + BHT and B20 + DEE, were 52.67, 72.68, 67.38, 65.35, 64.64, 66.03 and 70.75 kJ/m^3^, respectively. The curve of the diesel–PKO biodiesel blends showed the same pattern of heat release, irrespective of the applied loads [14]. The HRR for diesel was comparable to the B20, B30 and B100 PKO biodiesel blends. The B20 fuel blend recorded the second highest HHR due to better atomization of its low viscosity [26]. Results show that the B20BHT fuel blend showed 2% reduction in HHR (66.03 kJ/m^3^), compared with the B20 fuel blend, which could be due to the low level of oxygen content, whereas, the HRR of B20DEE increased by 4.7%, (70.75 kJ/m^3^) compared to the B20 fuel blend—this was almost close to that of diesel fuel. This result is in agreement with Imtenan et al. [21], who justified that the better atomization and fuel bound O_2_ content of DEE lead to complete burning, thereby increasing the HRR. However, late SOC was observed for the B20DEE blend, compared with the B20 blend (despite its higher cetane number), as shown in Figure 6. This could be because of higher vaporization of latent heat of DEE in the B20 blend, which causes injection into the low temperature zone [27,28].

#### 3.2.2. Cylinder Pressure

**Figure 7:**
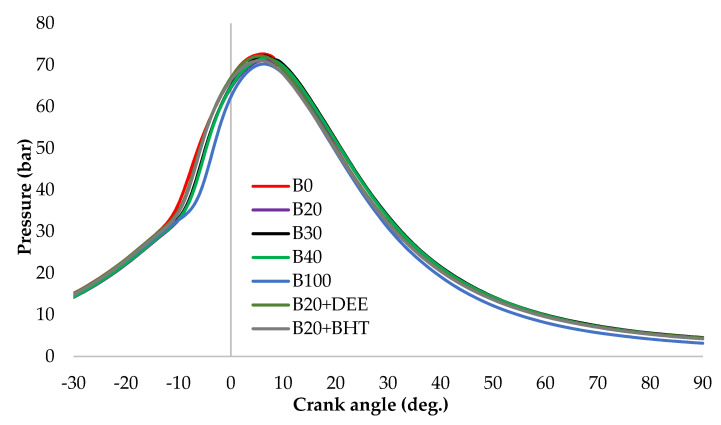
In-cylinder pressure for all fuel blends at full load.

Figure 7 depicts the in-cylinder pressure at full load condition of all fuel blends samples versus crank angle. Results show that the peak cylinder pressure of all fuel samples, B100, B0, B20, B30, B40, B20BHT, and B20DEE, happened within the range of 4–8° ATDC and was 69.04, 73.49, 72.73, 70.96, 70.12, 70.75 and 73.27 bar, respectively. The peak pressure rise showed similar patterns for all the fuels. The highest cylinder pressure (73.49 bar) was recorded for diesel fuel due to its high calorific value. The next highest pressure was obtained for the B20 fuel blend, at 72.73 bar, which was very close to that of diesel fuel. Among all the fuel samples, the lowest in-cylinder pressure was measured for B100, which could be due to the lower volatility and greater viscosity, which consequently leads to the low air-to-fuel mixture in the premixed burning zone, compared to diesel fuel [26]. Lower cylinder pressure was previously reported for biodiesel combustion compared to diesel fuel [29]. In addition, Elkelawy et al. [30] argued that this is because of the low self-ignition characteristics and heating contents of the biodiesel fuel, relative to diesel fuel [31]. The effect of adding the DEE additive into the B20 fuel blend slightly increased in-cylinder pressure by 1%, compared to B20. This could be attributed to the increased O_2_ content of the blend. The in-cylinder pressure of the B20DEE blend was slightly greater than that of the B20 blend, but lesser than that of diesel, probably due to the higher evaporation rate of DEE, resulting in lower cylinder temperature [16]. When adding BHT into B20, the pressure of the cylinder was reduced, compared with the B20 blend, due to the partial burning, because the BHT antioxidant reduces carbon oxidation (i.e., the OH radicals that participate in the combustion) by scavenging the OH radicals [17].

### 3.3. Performance Analysis

#### 3.3.1. Brake Thermal Efficiency (BTE)

**Figure 8:**
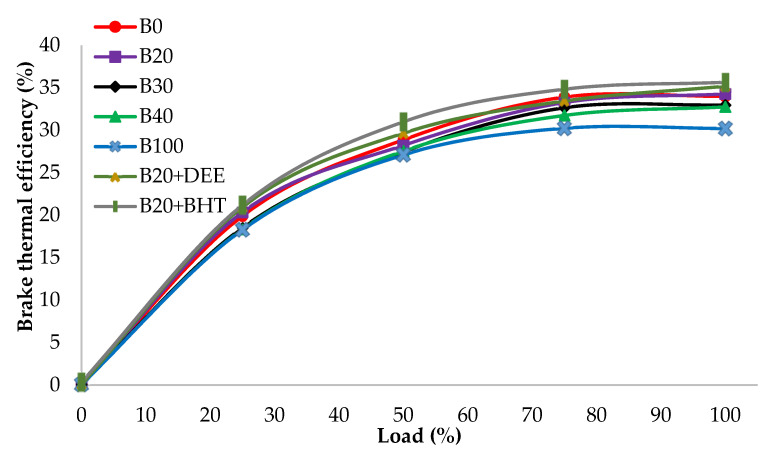
BTE of all fuel blends at different engine loads.

Figure 8 depicts the BTE of all tested fuel blends at various load conditions. BTE can be determined between the brake power output and the combustion fuel energy, which is one of the very important factors that tell us how expeditiously the heat energy is exchanged into effective work [32]. Brake thermal efficiency increased gradually with the load for all the fuel blends. As shown in Figure 8, diesel fuel recorded the maximum BTE, while the least BTE was found for B100 at 100% load. There are several reasons for this finding, such as higher viscosity (4.2 mm^2^/s), lower heating content and lower air-fuel mixing of biodiesel fuel [33]. The results show that the BHT additive could improve BTE at full load by 6.64% and 4.6%, compared to neat diesel and B20, respectively. Like all oxygenated additives, addition of DEE into the B20, resulted in higher BTE, compared to diesel and other blends of PKO biodiesel. The BTE increment of the B20DEE fuel blend was 5.4% and 3.38% more than B20 and diesel fuel, respectively. The reason might be due to the higher oxygen content of DEE, which contains 21.6% O_2_ in their molecule structure, and also to its low viscosity [27]. Increased BTE after adding oxygenated additive was reported by Ren et al., who examined several different oxygenated additives and observed that they could improve BTE generally [34].

#### 3.3.2. Brake Specific Fuel Consumption

**Figure 9:**
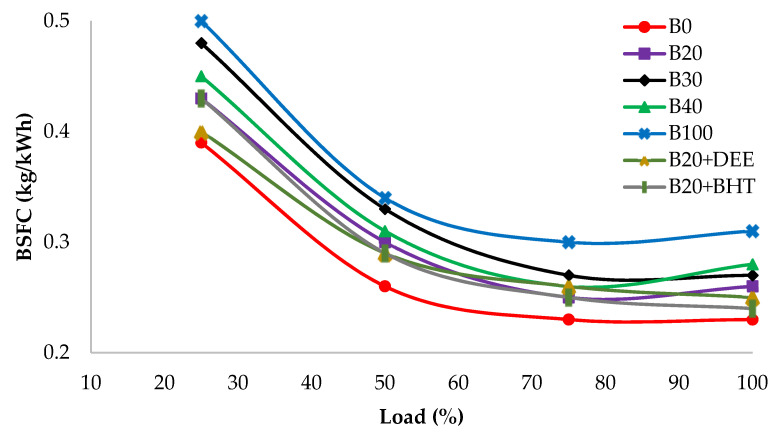
BSFC of all fuel blends at different engine loads.

BSFC is the mass rate of consumption of fuel per unit brake power, which is reciprocally proportional to BTE. Figure 9 shows the variation in BSFC of different fuel samples at constant engine speed and various load conditions. BSFC decreased with load increase, regardless of the fuel types [2,35]. Biodiesel showed an increase in BSFC, compared to diesel fuel. Tompkins et al. [36] argued that this is due to some reasons, such as higher viscosity, incomplete fuel burning and low CV of biodiesel fuel. These reasons are emphasized by other researchers [37,38]. At 100% load, B100 depicted the highest value, 0.31 kg/kWh of BSFC. On the other hand, diesel fuel recorded the lowest BSFC at 0.23 kg/kWh. However, the B20 fuel blend values were almost close to the diesel fuel ones at all type of loads [26]. After adding an oxygenated additive, the BSFC decreased, for the B20DEE fuel blend, by 0.25 kg/kWh, which was 3.84% lower than the B20 fuel blend. High fuel-bound oxygen content is the main reason which helps the DEE + B20 fuel blend to have better combustion efficiency. At low loads, the BSFC for DEE and diesel fuel was similar in value, probably due to the lower heat loss [17]. The effect of the BHT antioxidant additive recorded a BSFC of 0.24 kg/kWh, which was less than other diesel–biodiesel blends. However, this value was 7.69% lower than that of the B20 fuel blend and almost close to the diesel fuel one at full load. Similar results were reported by Vijay Kumar et al. for DEE combustion [39]. In addition, Kumar et al. reported that the addition of palm oil methyl ester into diesel fuel led to an increase in BSFC [40]. 

### 3.4. Emission Characteristics

#### 3.4.1. Nitrogen Oxides (NOx)

**Figure 10:**
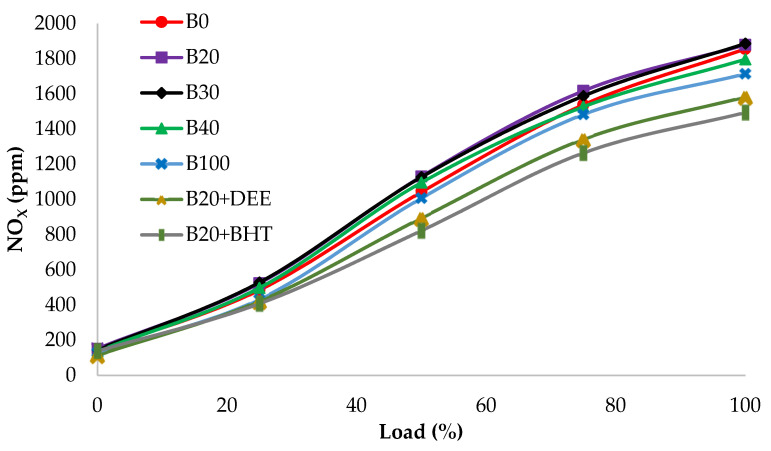
NOx emission of all fuel blends at different engine loads.

The NOx emission of all fuel tested at different loads are shown in Figure 10. NOx emissions increased steadily with load for all experimented fuel blends. NOx emission is formed through oxidation of nitrogen at high temperatures in the combustion chamber [31,41]. Therefore, NOx emission was at peak at full load compared to the low loads, because of higher flame temperature at higher loads [32]. 

From Figure 10, the results indicate that B20, B30 and B40 had a higher NOx emission than diesel fuel. This could be attributed to the biodiesel high molecule weight and also to the presence of high oxygen content, which increases the temperature locally, resulting in higher emissions of NOx [31,35]. The values of NOx emission of B100, B0, B20, B30, B40, B20BHT and B20DEE were 1714, 1855, 1879, 1886, 1796, 1494 and 1583 ppm, respectively. For the B20 blend, the NOx emission was slightly greater than diesel by 1.3%, whereas the NOx emission of B100 was 7.6% less than diesel fuel. The presence of DEE in B20 decreased NOx emission by 14.6%, compared to diesel fuel. This could be well justified by the higher latent heat of evaporation, which decreased the premixed combustion part, thereby resulting in combustion temperature reduction [17]. Among all fuel blends, the B20BHT fuel blend was found to have the lowest NOx emission, which was 19.4% less than that of diesel fuel. This could be due to the lower oxygen content, which leads to a decrease in cylinder combustion temperature [14]. In addition, BHT reduces free radicals formation, which subsequently results in lower NOx emissions [42].

#### 3.4.2. Carbon Monoxide (CO) 

**Figure 11:**
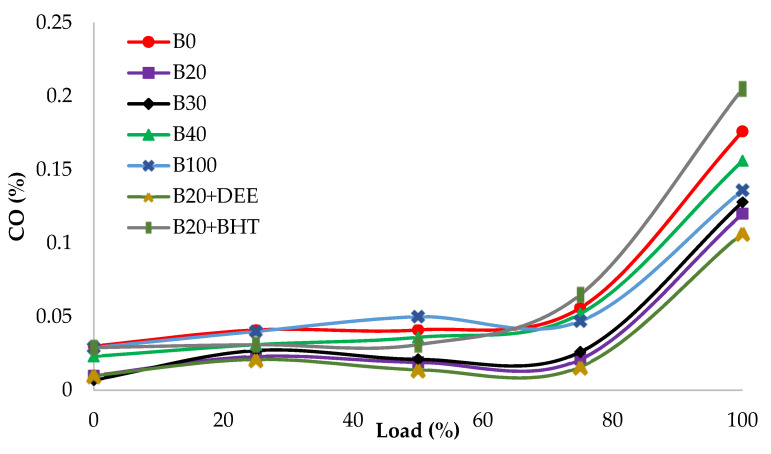
CO emissions of all fuel blends at different engine loads.

Figure 11 shows the CO emissions of different fuel blends at various loads. Carbon monoxide emission is mainly formed because of insufficient oxygen in the air/fuel mixture. CO emission results do not show significant variation at lower loads. However, at full load, rich fuel zones cause the incomplete combustion and CO emission drastically increases [43]. 

The measured values of CO emission for the fuel blends B100, B0, B20, B30, B40, B20 + BHT and B20 + DEE were 0.136, 0.176, 0.120, 0.128, 0.156, 0.205 and 0.107%, respectively. CO emission for all diesel–PKO biodiesel blends had minimum values, compared to the diesel fuel. This could be due to the high oxygen content, which provides more complete burning [1,44]. However, diesel had the highest CO emission (0.176%), compared to the blends of PKO biodiesel. CO emission of the B20 fuel blend does not show remarkable difference with diesel fuel (i.e., 0.12%). B100 and B40 recorded 11.7% and 23% higher CO emission than the B20 blend, but the measured value was less than that of diesel fuel [14]. The influence of DEE being added to B20 decreased CO emission by 10.8% and 39.2%, compared to B20 and diesel fuel, due to the higher amount of fuel bound oxygen content, which increases complete combustion even in locally rich fuel zones and subsequently leads more CO oxidation [17]. The high evaporation rate of DEE shortens the spray penetration length, thereby decreasing CO emissions [19]. As expected, the addition of the BHT antioxidant into B20 increased CO emissions by 14.14%, compared to diesel. Antioxidants have a high impact on scavenging OH radicals, which is directly related to the oxidation of CO to CO_2_ [42].

#### 3.4.3. Hydrocarbon (HC)

**Figure 12:**
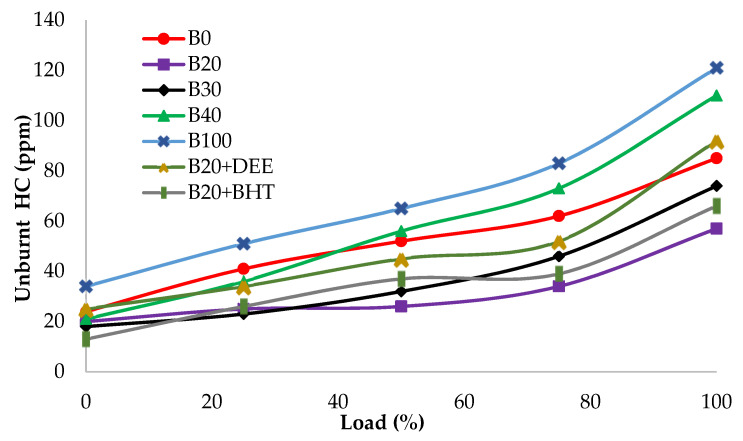
HC emissions of all fuel blends at different engine loads.

Figure 12 depicts the unburnt HC emissions of all tested samples at various engine loads. The main reason of HC emission formation is the poor dissipation of fuel in the rich fuel zones, which causes the partial or incomplete combustion. From the other side, in the lean air/fuel mixture it causes low cylinder temperature. In addition, HC emission is also due to the a thin layer of fuel on the combustion chamber wall, which are due to fuel impingement during over spray [30,38,45]. Furthermore, the trapping of fuel in the combustion chamber of crevice areas is also considered as one of the major factors for the increase in HC emissions [43].

All the fuel blends showed a steady increasing of HC emissions with load. The values of HC emissions for the different fuel tested, B100, B0, B20, B30, B40, B20BHT and B20DEE, were 121, 85, 57, 74, 110, 66 and 92 ppm, respectively. B40 and B100 had greater HC emission values, compared to diesel fuel, due to poor fuel dissipation in rich mixture zones, causing incomplete combustion because of the higher viscosity of biodiesel fuel [42,46]. Heywood has mentioned that higher fuel viscosity has important influence on the atomization of the fuel and, subsequently, the SOC, in the fuel burning process [20]. The HC emission was significantly decreased, for the B20 blend, by 32.9%, compared to diesel fuel. This could be attributed to the effective participation of oxygen molecules in the burning process, which leads to improved combustion quality [41,47]. On the contrary, the addition of additives to the B20 fuel blend showed increased emissions of HC, compared to the B20 fuel blend. At full load, the HC emission of the B20 + DEE blend was measured to be 38% higher than the B20 fuel blend. Apart from the higher oxygen content of DEE, the reason for the increase in HC emission of DEE blended in B20 might be the higher heat of evaporation rate, leans the outer part of the flame in the excess rich fuel zone in comparison with over-mixing of fuel with air. At higher loads, excess rich air-fuel ratio during combustion is a common phenomenon, which happens especially with oxygenated additives, such as DEE, which has low density and viscosity [27]. It is evident, from Figure 12, that, in the case of addition of 2000 ppm BHT antioxidant to the B20 fuel blend, HC emission was increased by 13.6%, compared to the B20 one. The reason for this increment is the negative effect of BHT on the oxidation of oxygen, which results in higher HC emissions [42].

#### 3.4.4. Smoke Opacity

**Figure 13:**
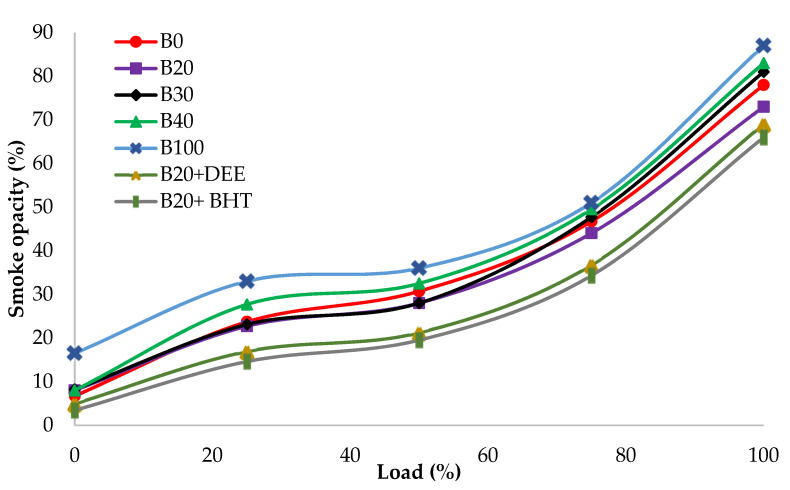
Smoke opacity of all fuel blends at different engine loads.

Smoke opacity is the measure of particulate emission (i.e., soot content) in the exhaust gas of a diesel engine. Smoke is mainly formed by incomplete fuel combustion because of variations in the operating conditions of the engine [48]. Smoke emission variations of all experimented fuel samples at various loads are depicted in Figure 13. The peak values of smoke emission, which were recorded at engine full load for B100, B0, B20, B30, B40, B20BHT and B20DEE, were 87, 78, 73, 81, 83, 66 and 69%, respectively. The smoke emission of all diesel–PKO biodiesel blends, except B20, were higher than those measured for diesel fuel. This is mainly because of poor biodiesel atomization, because of higher biodiesel viscosity which causes low in-cylinder temperature and, subsequently, incomplete combustion [26,49]. The intensity of smoke was increased with the increase in load and maximum values were observed at engine full load. During the combustion at higher engine loads, the thermal cracking of long chain unburned hydrocarbons in the insufficient oxygen happens, which leads to an increase in smoke emission [43]. 

Smoke emission was significantly decreased for the B20 blend, by 6.4%, compared to diesel fuel, which can be justified by the higher amount of oxygen molecules in the fuel rich zones. Generally, soot is formed mostly in the PC phase, where the stoichiometry air/fuel equivalence ratio prevails [35]. It can be seen, from Figure 13, that 5% DEE gave significantly lower smoke emissions, by 5.4% and 11.5%, compared to the B20 blend and diesel fuel, respectively. The presence of oxygen molecules in the chemical structures of DEE could enhance the quality combustion process [27]. The addition of the BHT antioxidant to the B20 blend also reduced smoke emissions by 9.5% and 15.4%, compared to the B20 fuel blend and diesel, respectively [14].

### 3.5. Energy Analysis

**Figure 14:**
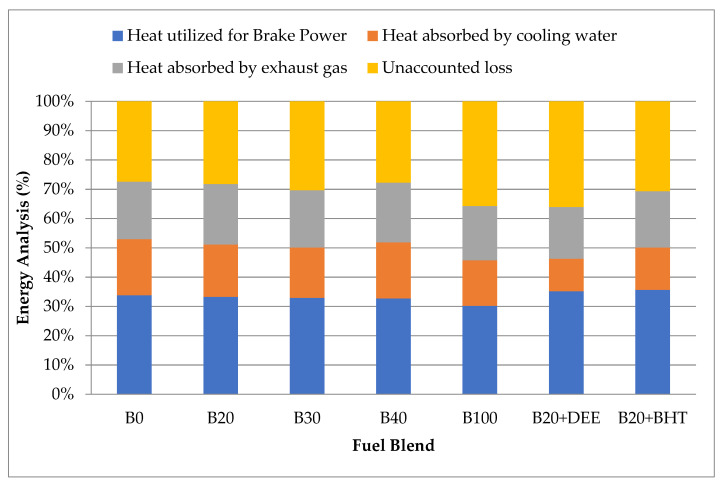
Energy analysis of all fuel blends at maximum engine loads.

The energy analysis results of brake power, cooling water, exhaust gas and unaccounted loss for the different fuel tested, B0, B20, B30, B40, B100, B20DEE and B20BHT are shown in Figure 14 at full loading conditions. A total of 33.78% of the fuel energy was converted into brake power, 19.16% was lost by the engine to the cooling water, 19.65% was lost through the exhaust gases and 27.41% of heat was exhausted by radiation for diesel fuel. Considering other fuels at the same working condition, it is seen that the conversion rate of fuel energy to brake power has a gradually decreasing trend. This ratio is the expression of thermal efficiency and was calculated as 33.27%, 32.96%, 32.74% and 30.2% for B20, B30, B40 and B100, respectively. These results show that operation with biodiesel blends leads to less brake power for the same fuel energy rate, since biodiesel has a lower heating value than diesel. Considering B20DEE and B20BHT, the conversion rates of fuel energy to brake power showed slight increases, compared to diesel fuel, which were 35.15% and 35.64%, respectively; this might be due to the higher oxygen content of fuel additives in their molecule structure and also to their low viscosity [50,51,52].

## 4. Conclusions

The present research explores the suitableness of diesel–PKO biodiesel blends as substitute fuel in DI diesel engines without any engine modifications. In addition, effects of two additives (DEE and BHT) on B20 were investigated. The optimization of PKO biodiesel production was successfully investigated experimentally and optimum yield conditions were also validated through MINITAB-17 and analyzed using the Taguchi method. The maximum biodiesel yields obtained using the KOH and NaOH catalysts were 86.69% and 75.21%, at the molar ratio of 6:1 and at the temperature of 55 °C, whereas the simulation designed by the MINITAB-17 software predicted the yields of 88% and 73.37%, respectively. Overall, the following conclusions can be drawn from the present study:

- The B20DEE blend attained 4.7% higher peak HRR than the B20 fuel blend, due to the better atomization and DEE.
- The B20BHT and B20 + DEE fuel blends showed higher BTE, by 6.64% and 5.4%, compared to B20, respectively.
- BSFC were decreased by 7.69% and 3.84% with the use of BHT and DEE additives, compared to the B20 fuel blend.
- NOx emissions were decreased by 19.4% and 14.6% in the presence of BHT and DEE, respectively, compared to diesel fuel, due to higher latent heat evaporation and lower premixed combustion phase.
- B20 showed a decrease in CO emission of 31.8%, compared to diesel fuel. However, adding DEE improved CO emissions by 39.2%, compared to diesel fuel.
- HC emission was increased by 13.6% and 38%, for B20BHT and B20DEE respectively, compared to the B20 fuel blend.
- B20DEE decreased smoke emissions by 5.4% and 11.5%, respectively, compared to B20 and diesel fuel, because of the higher oxygen content in the molecule structure of DEE.
- B20BHT combustion decreased smoke emissions by 9.5%, compared to the B20 fuel blend, because of the antioxidant’s influence on reducing ignition delay.

In generall, this experimental research provides a less polluted alternative fuel, which is more beneficial for our environmental concerns.

## Figures and Tables

**Table 1:** Range and levels of parameters.

Variables	Symbol	Range and Levels		
		−1	0	1
Temperature (°C)	T	50	55	60
Alcohol to molar ratio	MR	3:1	6:1	9:1
Catalyst	C		KOH	NaOH

**Table 2:** Properties diesel–PKO biodiesel blends and neat diesel fuel.

Properties	Standard Values	B100	B20	B30	B40	DIESEL	Testing Procedure
Density, 20 °C (kg/m^3^)	680–970	844	832	834	836	830	ASTM D1298
Kinematic viscosity, 40 °C (mm^2^/s)	1.9–6	4.2	3.12	3.28	3.39	2.75	ASTM D445
Flash point (°C)	60–190 °C	170	84	95	105	62	ASTM D93
Fire point (°C)	-	172	92	101	112	70	ASTM D93
Calorific value (MJ/kg)	-	38.2	42.68	42.12	41.56	43.8	ASTM D240
Cetane number (CN)	>40	52	-	-	-	53	ASTM D613

**Table 3:** Yield (%) of PKO biodiesel.

Catalyst	Temperature (°C)	Methanol Ratio	Yield (Experimental) (%)	Yield (Model) (%)
KOH	50	3:1	82.3215	79.6506
		6:1	85.6495	86.3099
		9:1	84.4795	86.0788
	55	3:1	83.4956	81.3492
		6:1	86.6935	88.0085
		9:1	85.7155	87.7774
	60	3:1	82.6175	80.0396
		6:1	86.0165	86.6989
		9:1	85.3915	86.4678
NaOH	50	3:1	62.3735	65.0131
		6:1	72.6465	71.6724
		9:1	72.6955	71.4413
	55	3:1	64.4395	66.7116
		6:1	75.2135	73.3710
		9:1	74.9999	73.1399
	60	3:1	62.9185	65.4021
		6:1	72.1025	72.0614
		9:1	73.4535	71.8303

**Table 4:** Engine specifications.

Make	Kirloskar (Direct Injection, Water Cooled, 1500 rpm)
Model	TAF 1 (5.2 kW power)
Ratio of compression	17.5:1
Bore × stroke (mm)	87.5 × 110 mm
Swept volume	661 cm^3^
Start of injection and pressure	24° BTDC and 21 MPa
Connecting rod length	234 mm

**Table 5:** Instruments uncertainty percentage and accuracy and its measuring range.

Instrument		Percentage Uncertainties	Measuring Range	Accuracy
AVL 3066A02 crank angle encoder		±0.3		±1
AVL pressure transducer GH12D		±0.01	0–250 bar	±0.01 bar
AVL DI GAS 444 N (five gas analyzer)	NOx	±0.5	0–5000 ppm vol	1 ppm vol
	O_2_	±0.35	0–25% vol	0.01% vol
	CO	±0.02	0–15% vol	0.0001 vol
	HC	±4 ppm	0–30,000 ppm vol	1 ppm/10 ppm
	CO_2_	±0.2	0–20% vol	0.1% vol
AVL 437C smoke meter	K-2 thermocouple	±0.3	(0–1250 °C)	±1 °C
	Smoke intensity	±1.1	0–100%	±1%
	U-tube manometer	±1.5		±1 mm
	Digital stopwatch	±0.3		±0.2 s
Burette		±1.5	1–30 cc	±0.2 cc

## Data Availability

Data sharing is not applicable to this article.

## Nomenclature

ATDC	after top dead center
B0	100% diesel fuel
B100	100% palm kernel oil biodiesel
B20	20% palm kernel oil biodiesel + 80% diesel fuel
B20 + BHT	B20 + butylated hydroxyltoluene
B20 + DEE	B20 + diethyl ether
B30	30% palm kernel oil biodiesel + 70% Diesel
B40	40% palm kernel oil biodiesel + 60% Diesel
BHT	butylated hydroxyltoluene
BP	brake power
BSFC	brake specific fuel consumption
BTDC	before top dead center
BTE	brake thermal efficiency
CI	Compression ignition
CN	cetane number
CO	carbon monoxide
CO_2_	carbon dioxide
DC	diffusion combustion
DEE	diethyl ether
FFA	free fatty acid
HHR	heat release rate
KOH	potassium hydroxide
NaOH	sodium hydroxide
NOx	nitrogen oxides
PC	premixed combustion
PKO	palm kernel oil
PO	palm oil
SOC	start of combustion
UHC	unburned hydrocarbon
WCO	waste cooking oil
